# The Nrf2/HO-1 Axis in Cancer Cell Growth and Chemoresistance

**DOI:** 10.1155/2016/1958174

**Published:** 2015-11-30

**Authors:** A. L. Furfaro, N. Traverso, C. Domenicotti, S. Piras, L. Moretta, U. M. Marinari, M. A. Pronzato, M. Nitti

**Affiliations:** ^1^Giannina Gaslini Institute, Via Gerolamo Gaslini 5, 16147 Genoa, Italy; ^2^Department of Experimental Medicine, University of Genoa, Via L. B. Alberti 2, 16132 Genoa, Italy; ^3^Bambino Gesù Children's Hospital, IRCCS, Piazza S. Onofrio 4, 00165 Rome, Italy

## Abstract

The transcription factor, nuclear factor erythroid 2 p45-related factor 2 (Nrf2), acts as a sensor of oxidative or electrophilic stresses and plays a pivotal role in redox homeostasis. Oxidative or electrophilic agents cause a conformational change in the Nrf2 inhibitory protein Keap1 inducing the nuclear translocation of the transcription factor which, through its binding to the antioxidant/electrophilic response element (ARE/EpRE), regulates the expression of antioxidant and detoxifying genes such as heme oxygenase 1 (HO-1). Nrf2 and HO-1 are frequently upregulated in different types of tumours and correlate with tumour progression, aggressiveness, resistance to therapy, and poor prognosis. This review focuses on the Nrf2/HO-1 stress response mechanism as a promising target for anticancer treatment which is able to overcome resistance to therapies.

## 1. Introduction

The availability of intracellular antioxidants is essential in maintaining redox homeostasis in living cells. In aerobic conditions, cells are constantly exposed to the generation of reactive oxygen species (ROS) that can impact proteins, lipids, and DNA, playing a pathological role in the development of various human diseases such as cancer [1]. Therefore, cells have evolved endogenous defence mechanisms so as to counteract oxidative stress and to maintain ROS at low physiological levels, and the redox sensitive transcription factor, nuclear factor erythroid 2 p45-related factor 2 (Nrf2), acts as a key regulator of antioxidant response, crucially involved in the preservation of the structure and the functioning of normal healthy cells [2–4]. However, cancer cells, differently from normal cells, show an increased rate of ROS generation as by-products of their metabolism [5] and, as “masters” of adaptation, they take advantage of the overactivation of antioxidant defences, in particular Nrf2-dependent genes [6–8]. This ability to adapt and survive under conditions of electrophilic, oxidative, and inflammatory stress is strongly dependent on the expression of a complex network comprising nearly 500 genes, induced by Nrf2, encoding proteins with different antioxidant and cytoprotective functions [9]. In particular, heme oxygenase 1 (HO-1) exerts a strong antioxidant and antiapoptotic effect favouring cancer cell growth and resistance to therapy. In this review, we focus our attention on the deleterious properties of Nrf2, and of its target gene HO-1, in relation to cancer cell growth and chemoresistance.

## 2. Nrf2: Structure and Regulation

The nuclear factor erythroid 2 p45-related factor 2 (Nrf2) is a transcription factor that plays a key role in the regulation of the cellular redox status. Indeed, Nrf2 controls not only the expression of antioxidants as well as phase I and phase II drug-metabolizing enzymes [10, 11], but also multidrug-resistance-associated protein transporters [12] (Table 1).

The human Nrf2 was first described, cloned, and characterised by Moi and coworkers in 1994 [13] and its cloned gene is encoded within a 2.2 kB transcript, predicting a protein of 589 amino acids with a molecular mass of 66.1 kDa [13].

**Figure 1:**
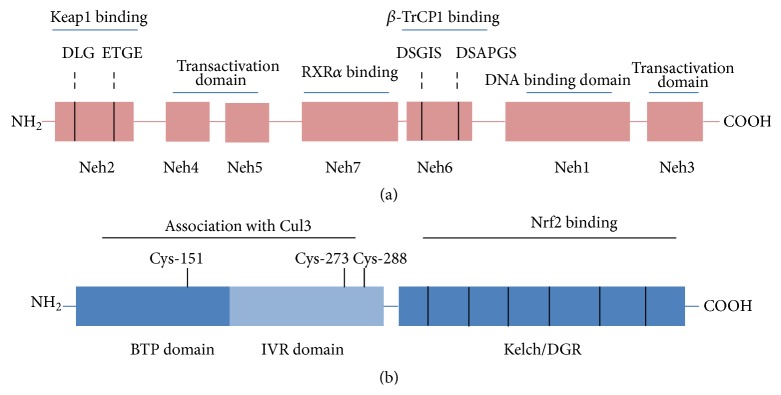
Schematic representation of Nrf2 and Keap1 structures. (a) Nrf2 contains seven domains, Neh1–Neh7. The Neh2 domain contains two binding motifs, DLG and ETGE, responsible for the interaction with Keap1. The Neh4, Neh5, and Neh3 domains are important for the transactivation activity of Nrf2. The Neh7 domain is critical for RXR*α* binding. The Neh6 domain regulates Nrf2 degradation by *β*-TrCP1. The Neh1 domain has a basic region leucine zipper motif for DNA binding. (b) Keap1 contains three major domains. The BTB domain mediates Keap1 homodimerisation and the IVR domain contains critical cysteine residues and together they associate with Cul3. The Kelch/DGR domain mediates the binding with the Neh2 domain of Nrf2.

Nrf2 has seven functional domains named Nrf2-ECH homology (Neh) 1–7 (Figure 1(a)) [12, 14, 15]. The Neh1 domain has a basic region leucine zipper structure needed for the dimerisation with small Maf and binds to antioxidant/electrophile responsive elements (ARE/EpRE) [16]. Neh2 is the main negative regulatory domain which binds to the Kelch-like ECH-associated protein 1 (Keap1) via the DLG and ETGE motifs [17]. Neh3 is localised in the C-terminal region of Nrf2 and acts as a transactivation domain recruiting the chromo-ATPase/helicase DNA binding protein family (CHD6) [6], whereas both Neh4 and Neh5 are transactivation domains that recruit cAMP response element binding protein- (CREB-) binding protein (CBP) and/or the receptor-associated coactivator (RAC) [18]. The Neh6 domain mediates the Keap1-independent degradation of Nrf2 through recruitment by the DSGIS and DSAPGS motifs of the dimeric *β*-transducin repeat-containing protein (*β*-TrCP) [19]. Lastly, the Neh7 domain, recently identified by Wang and coworkers, interacts with the retinoid X receptor alpha (RXR*α*), a repressor of Nrf2 [20].

### 2.1. Keap1-Dependent Posttranscriptional Regulation

#### 2.1.1. Ubiquitination and Proteasomal Degradation of Nrf2

Under basal conditions, Nrf2 is localised in the cytosol associated with its negative regulator Keap1, an adaptor component of Cullin 3-based ubiquitin E3 ligase complex (Cul3) that promotes Nrf2 constant ubiquitination and proteasomal degradation, maintaining low basal levels [21, 22]. Nrf2 turnover is rapid, less than 20 minutes, and prevents the expression of Nrf2 target genes under normal conditions [23]. On exposure to oxidative or electrophilic stress, Keap1 is modified whereas the enzymatic activity of the E3 ubiquitin ligase is inhibited; Nrf2 is liberated from Keap1, accumulates in the nucleus, dimerises with small Maf protein, and then activates, after ARE-sequence binding, the transcription of its target genes [23, 24]. Thus, Keap1 is the main repressor of Nrf2, having three functional domains, namely, the broad complex, Tramtrack, and Bric-a-Brac (BTB) domain, the intervening region (IVR) domain, and the double glycine repeat (DGR)/Kelch domain [25] (Figure 1(b)).

**Figure 2:**
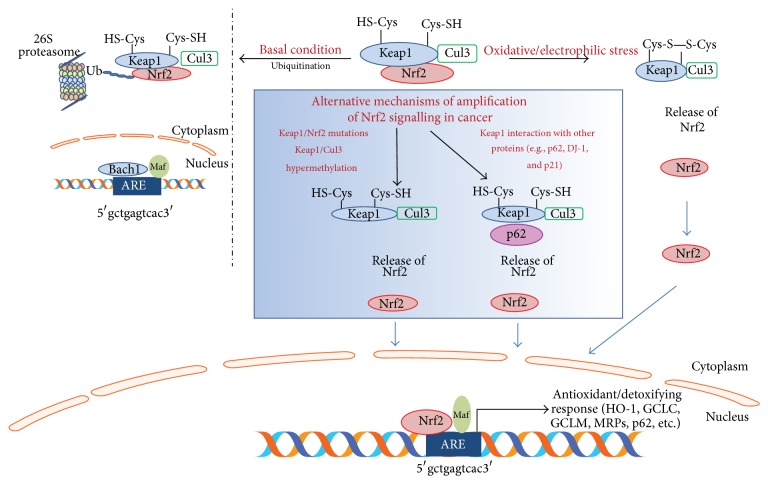
Nrf2 activity regulation. In a resting state, Nrf2 is sequestered in the cytoplasm through the binding with Keap1, responsible for Nrf2 ubiquitination and proteasomal degradation via Cul3. Oxidative/electrophilic stress causes a conformational change in Keap1-Cul3, by acting on specific cysteine residues in Keap1, leading to Nrf2 dissociation. Thus, free Nrf2 translocates to the nucleus, which dimerises with small Maf protein and binds to ARE/EpRE sequence within regulatory regions of a wide variety of target genes (e.g., HO-1, GCLC, GCLM, MRPs, and p62). In cancer cells (blue box), Keap1/Nrf2 mutations and Keap1/Cul3 aberrant hypermethylations as well as Keap1 interactions with ETGE motif-containing proteins lead to an increased Nrf2 activation and induction of target genes.

Keap1 acts as a sensor for oxidative and electrophilic stress through the modification of 27 cysteine residues [26] (Figure 2). The main three cysteine residues for the regulation of Nrf2 activity are Cys151 in the BTB domain and Cys273 and Cys288 in the IVR domain, all of which are targets of oxidative and electrophilic modifications [27–29]. It has been shown that cells expressing Keap1 Cys151 point mutant protein show reduced activation of Nrf2 in response to a number of inducers (e.g., sulforaphane, tert-butylhydroquinone, and diethyl maleate) in comparison to the wild-type cells [30] and that Cys273 and Cys288 are critically required for the basal repression of Nrf2 [31]. In addition, the modification of a subset of cysteine residues in Keap1, by Nrf2 inducers, supports the hypothesis of a “cysteine code” which is critical in the activation of Nrf2 [32].

#### 2.1.2. Autophagic Degradation of Keap1

Furthermore, other interacting protein partners, such as the sequestosome-1 protein (p62/SQSTM1), can modulate Nrf2 activity [33, 34]. p62/SQSTM1 is a scaffold protein that binds to polyubiquitinated proteins and targets protein aggregates for autophagic degradation. p62 contains an STGE-binding motif similar to the Nrf2 ETGE motif [33] which is needed for the direct interaction with Keap1 [35]. As a consequence, Keap1 is sequestered in autophagosomes and Nrf2 ubiquitination decreases, leading to a prolonged activation of Nrf2 in response to oxidative stress [34, 35] (Figure 2).

### 2.2. Keap1-Independent Posttranscriptional Regulation

Recently, alternative mechanisms for the degradation of Nrf2 have been identified. For example, a number of studies have demonstrated that glycogen synthase kinase 3*β* (GSK-3*β*) directs the ubiquitination and proteasomal degradation of various transcription factors, including Nrf2, through the activation of E3 ubiquitin ligase complex (*β*-TrCP-Skp1-Cul1-Rbx1) [19, 36]. Indeed, GSK-3*β* is able to phosphorylate Ser residues located in the Neh6 domain of Nrf2 which are then recognised by *β*-TrCP and, through the binding to Cullin 1 (Cul1) scaffold protein, lead to Nrf2 ubiquitination and degradation in a redox-independent manner [19, 37].

Moreover, a novel E3 ubiquitin ligase, namely, Hrd1, has been described by Wu and coworkers [36]. They showed that Hrd1 controls the Nrf2 stability by way of an interaction between the C-terminal domain of Hrd1 and the Neh4-5 domains of Nrf2. This Hrd1-mediated ubiquitination of Nrf2 is independent of both Keap1 and *β*-TrCP and compromises the Nrf2-mediated protection during liver cirrhosis [36].

### 2.3. Nrf2 Regulation at the Transcriptional Level

Although strong evidence shows that Nrf2 is primarily regulated at the protein level, it has also been demonstrated that the oncogenic KRAS transcriptionally upregulates the mRNA levels of Nrf2 through a TPA response element (TRE) located within the Nrf2 promoter. The oncogenic mutation of KRAS, or KRAS overexpression, indeed, is able to activate the Nrf2-dependent pathway [38]. In addition, an increase in Nrf2 mRNA levels has been shown to occur in response to the oncogenic activation of BRAF and C-MYC [39].

## 3. Nrf2 and Chemoprevention

It is well known that Nrf2 plays a key role in the cellular adaptation and protection against oxidative stress. The ability of Nrf2 to activate cytoprotective genes, which code for detoxifying enzymes, drug transporters, antioxidants, and anti-inflammatory proteins, plays a crucial role in reducing electrophiles and ROS, thus decreasing DNA damage and mutations and preventing genomic instability in normal cells. Several studies have shown that, in Nrf2 null mice, there is an enhanced susceptibility to chemical carcinogens, such as benzo(a)pyrene, compared to wild-type mice, due to a decreased expression of phase II detoxification and antioxidant enzymes [40]. In a similar way, after exposure to N-nitrosobutyl (4-hydroxybutyl) amine (BBN), Nrf2 knockout mice develop urinary bladder carcinoma [41] and show an increased incidence of skin, colorectal, and mammary tumours [42–44]. Moreover, the protective role of Nrf2 against carcinogenesis is highlighted from studies in humans on single-nucleotide polymorphism (SNP) in the promoter region of the Nrf2 gene [45]. As described by Suzuki and coworkers, A/A homozygotes of Nrf2 rSNP-617 showed decreased expression of Nrf2 and consequently an increased risk of developing lung cancers, especially in the case of individuals with smoking habits [46].

## 4. The Dark Side of Nrf2 in Cancer Biology

Several studies have shown a deleterious aspect of Nrf2 defined as the “dark side of Nrf2” [47]. Its high and prolonged activation in cancer cells has been long associated with progression, metastatic invasion, angiogenesis, and chemo- and radio-resistance in tumours and is considered a poor prognostic factor [15, 48]. Indeed, the stable overexpression of Nrf2 was found in various types of tumours such as lung [49, 50], breast [51], head and neck [52], ovarian [53], and endometrial cancer [54].

### 4.1. Molecular Mechanisms of Nrf2 Activation in Cancer Cells

Several mechanisms have been shown to be involved in the constitutive activation of Nrf2 in cancer cells (Figure 2), mainly gain-of-function mutations in Nrf2 and loss-of-function mutations in Keap1, leading to an impairment of the binding to Keap1. This results in the stabilisation of Nrf2 and in the activation of its target genes, as identified in patients with lung or head and neck cancer [55, 56], whereas the loss-of-function mutations of Keap1 are mainly located in the Kelch/DRG domain and in the IVR domain [14] as observed in gastric, hepatocellular, colorectal, lung, breast, and prostate carcinomas, as reviewed by Shelton and Jaiswal [57].

In addition, epigenetic modifications in Keap1 are responsible for the accumulation and activation of Nrf2. Aberrant hypermethylation, inhibiting Keap1 gene expression, results in the accumulation of Nrf2, as shown in lung and malignant glioma [58], prostate [59], and colorectal cancer [60]. Moreover, somatic mutations and hypermethylation in CUL3 have been identified as being responsible for Nrf2 activation [53, 61, 62], as shown in thyroid, head and neck, and ovarian cancers [63–65].

### 4.2. Nongenomic Alteration of Nrf2 in Cancer Cells

Increased levels of Nrf2 in cancer cells can also occur in the absence of genomic alterations. In fact, much evidence shows that different proteins can alter the Nrf2-Keap1 binding [14]. Nrf2 activity is subject to a positive regulation by p21 [66, 67], which interferes with Keap1-mediated ubiquitination, interacting with the DLG motif in Nrf2, leading to its stabilisation. Therefore, Nrf2 expression is significantly lower in the absence of p21, and conversely it is increased upon p21 overexpression [67].

It is a fact that DJ-1, a protein belonging to the Thi/PfpI superfamily, is able to stabilise Nrf2 by preventing its association with Keap1, thus reducing ubiquitination and subsequent proteasomal degradation [68, 69]. DJ-1 in cancer is often overexpressed and leads to increased detoxification enzymes, such as NQO1, providing a survival advantage [68, 70, 71].

As previously described, p62 sequesters Keap1 in autophagosomes, leading to Nrf2 activation [72, 73]. Moreover, it has been shown that p62 contains the ARE sequence in its promoter region which is responsible for its Nrf2-dependent induction in response to oxidative stress, thus generating a positive feedback loop [73].

The list of proteins which can interact with Nrf2 and Keap1 and, therefore, modulate their regulation is continuously expanding (e.g., WTX, PALB2, and DPP3) [74–76]. All of these proteins contain an ETGF motif, suggesting that they are capable of upregulating Nrf2 by competing for Keap1 binding and suppressing Keap1-mediated ubiquitination of Nrf2 [14].

### 4.3. Nrf2 Activation and Hallmarks of Cancer Malignancy

Different Nrf2 target genes are associated with cancer cell proliferation and death. Among these, those genes involved in the pentose phosphate pathway such as glucose-6-phosphate dehydrogenase, phosphogluconate dehydrogenase, transketolase, and transaldolase 1 are responsible for NADPH and purine regeneration and, therefore, accelerate cancer cell proliferation [14]. Nrf2 may also bind to the ARE sequence in the promoter region of Notch1, contributing directly to its expression and leading to a more malignant phenotype and a more aggressive growth [77]. Moreover, Nrf2 is directly involved in the basal expression of the p53 inhibitor Mdm2, through the binding to the ARE sequence located in the first intron of this gene. It has been demonstrated that, in Nrf2-deleted murine embryonic fibroblasts (MEFs), Mdm2 expression is repressed and, compared to wild-type cells, a high level of p53 is accumulated, favouring cell death [78].

In addition, elevated levels of Nrf2 have been observed in various tumours with high metastatic potential [79], characterised by epithelial-mesenchymal transition (EMT) and the degradation of extracellular matrix (ECM), exerted by metalloproteases (MMPs). A major step of EMT is the loss of E-cadherin and the gain of N-cadherin [80]. It has been shown that E-cadherin is able to bind to the C terminus of Nrf2, preventing its nuclear accumulation, and that, during EMT, the overexpression of N-cadherin reduces the Nrf2 inhibition, thus favouring its activity [81]. Moreover, the knockdown of the Nrf2 by short hairpin RNA (shRNA) in esophageal squamous cell carcinoma (ESCC) suppressed the expression of MMP-2 and enhanced E-cadherin mRNA levels, resulting in a decreased invasion and migration of cancer cells [82].

Furthermore, the upregulation of Nrf2 is also related to angiogenesis which is promoted by HIF-1*α*, a transcription factor that senses oxygen homeostasis and is deregulated in tumours in hypoxic environments [83]. Under hypoxic conditions, indeed, the O_2_-dependent regulator prolyl-hydrolase domain (PHD) is catalytically inactive and increases the stability of HIF-1*α*. Consequently, the expression of its target proteins, including the vascular endothelial growth factor (VEGF), is enhanced [84]. It has been shown that Nrf2 silencing blocks HIF-1*α*-dependent VEGF expression in HT29 colon cancer and suppresses tumour growth with a concomitant reduction in VEGF-induced angiogenesis in mouse xenograft models [84].

### 4.4. Nrf2 and Cancer Resistance to Therapies

Several studies have shown that cancer cells with high levels of Nrf2 are less sensitive to etoposide, cisplatin, and doxorubicin [14]. Doxorubicin-resistant human cancer cells, such as ovarian SKOV3 and OV90 and mammary MCF-7/DOX, have shown high levels of Nrf2-ARE binding and ARE-driven luciferase activity, as well as the upregulation of Nrf2 target genes compared to the respective sensitive cell lines A2780 and MCF-7 [85, 86]. Non-small cell lung carcinoma A549 cells, in which Nrf2 is strongly activated, have shown a higher resistance to cisplatin compared to NCI-H292 and LC-AI cells [87]. Furthermore, Jayakumar and coworkers have demonstrated the role of Nrf2 and its dependent genes in the radioresistance of prostate tumour cells. Specifically, the radioresistant DU145 cells show enhanced levels of Nrf2 and a high GSH/GSSG ratio in comparison to the radiosensitive PC3 cells which show a faster depletion of GSH after radiation exposure [88]. It has also been shown that radiotherapy significantly reduces cancer cell survival when applied in combination with the Nrf2 inhibitor 4-(2-cyclohexylethoxy)-aniline, IM3829 [89]. Moreover, our own studies demonstrate that Nrf2 activation plays a key role in the resistance of neuroblastoma cells to GSH depletion or proteasome inhibition [90, 91].

In addition, Nrf2 activity is related to the upregulation of several multidrug-resistant efflux pumps, such as the ATP-binding cassette, subfamily G, member A2 (ABCG2), which favours drug resistance. It has been shown, indeed, that Nrf2 silencing attenuates the expression of the ABCG2 transcript and protein and sensitises lung cancer cells to mitoxantrone and topotecan, two representative chemotherapeutic drugs effluxed mainly by the presence of ABCG2 [92]. Multidrug-resistant protein-3 (MRP3), MRP4, and MRP5 are all upregulated by Nrf2 [93, 94]. Upregulation of MRP3 and GSTs leads to the increased hydrophilicity and excretion of cytotoxic agents, such as cisplatin, etoposide, and doxorubicin [95].

Recently, it has been proven that Nrf2 also plays a critical role in the drug resistance of cancer stem cells [96–98]. The resistance of glioblastoma is due to the presence of glioblastoma stem cells (GSCs) which confer tumourigenic potential and a survival advantage against chemotherapy [99]. Moreover, it has been shown that while knocking down Nrf2 decreases the self-renewing activity of GSCs [100, 101], the enhancement of Nrf2 levels and of its downstream genes, that is, HO-1, GCLM, and NQO1, is related to increased tumourigenic activity of the human mammospheres in comparison to their adherent counterparts, MCF-7 and MDA-MB-231 cells [102].

## 5. HO-1 as a Key Effector of Nrf2 Upregulation in Tumour Progression

**Figure 3:**
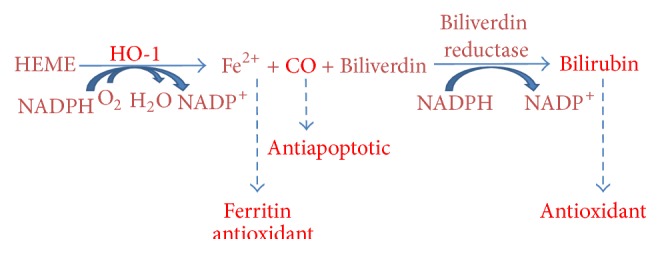
Heme catabolic pathway. HO-1 catalyses the degradation of heme into biliverdin/bilirubin (antioxidant), carbon monoxide (antiapoptotic), and ferritin (antioxidant) induced by free iron release.

Heme oxygenase 1 (HO-1) is considered one of the main effectors of Nrf2-dependent cell responses [48]. HO-1 is the inducible form of heme oxygenase, the first rate-limiting enzyme in the degradation of heme into biliverdin/bilirubin, carbon monoxide (CO), and ferritin induced by free iron release [103–105] (Figure 3). It is a 32 kDa stress protein present at low levels in most mammalian tissues [106] and its expression is induced by a wide variety of stress stimuli, including its substrate, as well as heavy metals [107], UV irradiation, ROS [108], nitric oxide [109], and inflammatory cytokines [110]. HO-1 and its metabolic products are involved in the maintenance of cellular homeostasis and they play a key role in the adaptive response to cellular stress as well as in the protection of healthy cells, preventing them from being transformed into neoplastic cells by counteracting ROS-mediated carcinogenesis [111–115].

However, HO-1 has been widely recognised as playing an important role in the malignant transformation of cancer cells. High levels of HO-1 have been found in various human tumours, inducing survival advantage, aggressiveness, and poor outcome [116–122]. HO-1 overexpression has been considered to be involved in invasive and metastatic mechanisms [123–125], and “in vitro” and “in vivo” studies, including clinical data, have shown that the inhibition or silencing of HO-1 inhibits this behaviour [125–128]. Moreover, a proangiogenic role of HO-1 in cancer has been reported “in vitro” and “in vivo” [123, 124, 127–130]. Finally, HO-1 has been shown to be correlated with resistance to chemo-, radio-, and photodynamic therapies [116, 119, 131–136] and its inhibition is able to sensitize cancer cells to death [90, 91, 137–139].

However, the role of HO-1 in cancer biology is not completely understood and some disputes in literature remain about its role in tumour progression, especially with regard to different types of tumours. It should be taken into consideration that several studies have reported that HO-1 activation prevents breast cancer proliferation [140] and prostate cancer angiogenesis [141] and mediates the anticancer activity of some drugs such as andrographolide by reducing the MMP-9 expression in breast cancer cells [142]. Moreover, considering the complex cross talk between HO-1 activity and cellular metabolic pathways, reviewed in depth by Wegiel and coauthors [143], it would seem conceivable that HO-1 can be subject to different modulations in different tumours, since the various metabolic statuses of cancer cells may influence how HO-1 activity modulates tumour growth. Therefore, it is important to note that HO-1 expression is controlled by other transcription factors other than Nrf2. Indeed, specific consensus sequences for both NF-kB and AP-1 are present in the promoter region of HO-1 [144–148] which, then, may be activated in response to different stimuli through the activation of different intracellular signaling pathways, as widely reviewed in [136, 149–151], suggesting a highly complex regulation which, up to now, is far from being fully understood.

However, what seems most interesting is the association between the upregulation of Nrf2 and the activation of HO-1 in tumour progression which correlates with cancer aggressiveness and malignancy. Interestingly, for instance, in human samples from gallbladder cancers, the upregulation of both Nrf2 and HO-1 correlates with tumour aggressiveness and a poor clinical outcome [79]. Moreover, Nrf2/HO-1 association has been widely reported, for instance, in non-small lung cancer, cervical cancer, hepatoma [152], esophageal squamous carcinoma [82], and multiple myeloma [153] (Table 2). In particular, malignant transformations have been associated with Nrf2-dependent HO-1 activation in B lymphocytes exposed to prostaglandin J2 [154]. Therefore, the gain of metastatic phenotypes is correlated with the overexpression of Nrf2 in association with the activation of HO-1, as shown in osteopontin-induced glioma cell invasiveness [155]. Furthermore, resistance to therapies has been related to the activation of Nrf2 together with HO-1, as shown by our group in neuroblastoma cells after GSH depletion or bortezomib exposure [90, 91] and by others in cisplatin-treated ovarian carcinoma cells [156] and in doxorubicin-resistant breast cancer cells [86].

Therefore, HO-1 activation provides tumour cells with strong survival advantage exerted by the antioxidant and antiapoptotic properties of its metabolic products. Moreover, when HO-1 activation is dependent on Nrf2 activity, generally this leads to highly aggressive cancer phenotypes. This can also account for the parallel activation of other Nrf2-dependent genes which can contribute to the prosurviving effect, for instance, by inducing HIF*α* [82], increasing multidrug-resistance-related proteins [79], or increasing the synthesis of GSH [8]. Unpublished data from our laboratory show that HO-1-dependent bilirubin generation and increasing amounts of GSH are key factors in inducing resistance to bortezomib in high-risk neuroblastoma.

## 6. Pharmacological Modulation of Nrf2/HO-1 Axis as a Strategy in Anticancer Treatment

The role of Nrf2/HO-1 axis in protecting cells by overcoming environmental stresses has already been demonstrated and compounds that are able to modulate this activity are well worth being considered. The activation of this pathway in normal cells can prevent tumour formation while its inhibition can be useful in improving cancer therapies.

As far as cancer prevention is concerned, different activators of Nrf2 have been proposed and have been intensively reviewed over the past 10 years [42, 157–166]. Many of these compounds are plant-derived phytochemicals, such as sulforaphane, curcumin, epigallocatechin-3-gallate, resveratrol, cinnamoyl-based compounds, garlic organosulfur compounds, and lycopene. These molecules have been considered to be chemopreventive due to their ability to induce antioxidant/detoxification enzymes, including HO-1, and xenobiotic transporters, through the activation of Nrf2 [18, 41, 50, 167–170]. On the other hand, the pharmacological inhibition of Nrf2/HO-1 axis has also recently emerged as a promising approach for cancer therapy.

Starting from the modulation of HO-1, the major effector of the pathway considered, several “in vivo” studies have confirmed the usefulness of the HO-1 competitive inhibitor zinc (II) protoporphyrin IX, ZnPPIX, in the reduction of hepatoma, sarcoma, lung cancer, and B-cell lymphoma growth in mice [129, 171]. Moreover, the PEG conjugation of ZnPPIX, which increases water solubility of the inhibitor, is able to improve its clinical application [172]. Another highly water-soluble micellar form of ZnPPIX, the amphiphilic styrene maleic acid copolymer (SMA-ZnPPIX) with a potent antitumour activity both “in vitro” and “in vivo,” has been additionally proposed [173].

However, it is important to note that pharmacological HO-1 inhibitors, as well as HO-1 activators, are responsible for strong HO-1 independent activities due to some nonspecific properties of these compounds [174, 175] and therefore the employment of siRNA could be more specific. Indeed, this approach is able to induce apoptosis of colon carcinoma cells [176, 177] and to diminish proliferation, growth, and angiogenesis in orthotopic hepatocellular tumours in mice [127].

As already discussed in the previous paragraph, there is also some evidence that HO-1 activation, induced by cobalt protoporphyrin IX (Co PPIX) or heme [140], as well as the overexpression of HO-1 can block tumour growth and invasion in “in vitro” studies, and this seems to be dependent on the cancer cell type and experimental model used.

Furthermore, the inhibition of Nrf2, acting upstream from HO-1 activation and involving also other downstream targets in addition to HO-1, could be a successful therapeutic approach.

Unfortunately, only a few inhibitors of Nrf2 have been developed so far. Among them, brusatol, extracts from* Brucea javanica* growing in Southeast Asia and Northern Australia, is able to decrease Nrf2 protein levels as well as decreasing the expression of its target genes, thus enhancing the cytotoxic effect of several chemotherapeutic agents, both “in vitro” and “in vivo” [178, 179].

In addition, flavonoid luteolin (3′,4′,5,7-tetrahydroxyflavone) found at high concentrations in celery, green pepper, parsley, perilla leaf, and chamomile tea has been shown to be another strong and selective Nrf2 inhibitor, which is able to reduce the constitutive expression of NQO1 in HepG2, Hepa1c1c7, and RL-34 cells in a time- and dose-dependent manner [180]. At physiological concentrations, luteolin inhibits Nrf2 activity by enhancing Nrf2 mRNA turnover, and it has been shown to sensitise NSCLC A549 cells to therapeutic drugs [181]. Similar results have been observed in the sensitisation of colorectal cancer cell lines to oxaliplatin “in vitro” [182] and “in vivo” in the chemotherapy of non-small cell lung cancer, NSCLC. Moreover, its oral administration, either alone or combined with an intraperitoneal injection of cisplatin, is seen to greatly inhibit the growth of xenograft tumours from the NSCLC cell line A549 in athymic nude mice [183].

Furthermore, all-trans-retinoic acid (ATRA) is able to suppress the Nrf2 pathway [184]. ATRA like other agonists of RA receptor *α* (RAR*α*) and retinoid X receptor *α* (RXR*α*) was shown to inhibit the basal and the inducible activity of Nrf2 both “in vitro” and “in vivo” [12, 185]. After ATRA treatment, Nrf2 forms a complex with RAR*α*. This complex is unable to bind to the ARE sequences, thus decreasing the ability of Nrf2 to activate its target genes [15]. In acute promyelocytic leukaemia cells, the cytotoxic drug arsenic trioxide (ATO) induces an antioxidant response characterised by Nrf2 nuclear translocation and enhances the transcription of its downstream target genes such as HO-1, NQO1, GCLM, and ferritin. It has been shown that, after cotreatment of ATO plus ATRA, the Nrf2 nuclear translocation is prevented and the cytotoxic effects of ATO treatment are enhanced [186].

Lastly, a recent paper shows that metformin, which was previously associated with a better survival of diabetic patients with pancreatic cancer [187], exerts its antitumour activity by suppressing HO-1 expression in cancer cells. In this paper, metformin is reported to inhibit Nrf2 through a Keap1-independent mechanism by inactivating Raf and ERK signaling [152]. The excellent therapeutic index of metformin, with few side-effects associated even with long-term treatment, could increase the chances of its application in cancer therapy.

## 7. Conclusions

The activation of Nrf2/HO-1 axis plays a central role in cellular adaptive responses to oxidative stress and cytotoxic insults representing a crucial point in the prevention of carcinogenesis. On the contrary, in tumour tissues, a prolonged activation of Nrf2 and HO-1 contributes to the gain of malignant phenotypes. Consequently, the Nrf2/HO-1 axis can be used by cancer cells to promote their growth advantage, metastatic potential, and resistance to therapy. Therefore, the therapeutic usefulness of inhibitors of Nrf2, and of its target gene, HO-1, especially in combination with conventional antineoplastic therapies, may well represent a potential and promising approach in the fight against cancer.

## Figures and Tables

**Table 1:** Genes regulated by Nrf2 in mice and humans.

Symbol	Name	Species	References
Antioxidant genes			
GCLC	Glutamate-cysteine ligase, catalytic subunit	m, h	[188, 189]
GCLM	Glutamate-cysteine ligase, modifier subunit	m, h	[188, 189]
GLRX	Glutaredoxin 1	h	[190]
GPX2	Glutathione peroxidase 2	m, h	[190, 191]
GPX4	Glutathione peroxidase 4	m	[16]
GSR1	Glutathione reductase	m, h	[188, 190]
SLC6A9	Glycine transporter	m	[16]
SLC7A11	Cysteine/glutamate transporter	m, h	[16, 188]
PRDX1-6	Peroxiredoxins 1 and 6	m, h	[10, 16]
SRXN1	Sulfiredoxin-1	m, h	[11, 188]
TXN1	Thioredoxin	m, h	[10, 16]
TXNRD1	Thioredoxin reductase 1	m, h	[188, 190]

HO-1-related genes			
HMOX1	Heme oxygenase 1	m, h	[10, 188]
BLVRA	Biliverdin reductase A	h	[190]
BLVRB	Biliverdin reductase B	m, h	[16, 190]
FECH	Ferrochelatase	h	[188]
FTH1	Ferritin, heavy polypeptide 1	m, h	[10]
FTHL12-17	Ferritin, heavy polypeptides 12 and 17	h	[188, 190]
FTL1	Ferritin, light polypeptide	m, h	[188, 190]

Detoxifying enzymes			
NQO1	NAD(P)H:quinone oxidoreductase 1	m, h	[188, 190]
GSTA1	Glutathione S-transferase class Alpha 1	m	[192]
GSTM1	Glutathione S-transferase class Mu 1	m	[192]
GSTP1	Glutathione S-transferase class Pi 1	m	[12]
UGT1A1	UDP glucuronosyl- transferase 1 family	h	[190]
UGT2B7	UDP glucuronosyl- transferase 2 family	m, h	[16, 190]

Drug transporters			
ABCB6	ATP-binding cassette, subfamily B(MDR/TAP)	m, h	[10, 11]
ABCC1	ATP-binding cassette, subfamily C(CFTR/MRP)	m	[193]
ABCC2	ATP-binding cassette, subfamily C(CFTR/MRP)	m, h	[193]
ABCC3	ATP-binding cassette, subfamily C(CFTR/MRP)	m, h	[193]
ABCC4	ATP-binding cassette, subfamily C(CFTR/MRP)	m	[193]
ABCC5	ATP-binding cassette, subfamily C(CFTR/MRP)	m	[194]

**Table 2:** Nrf2 and HO-1 upregulation in tumours.

Type of tumor	Nrf2	HO-1	Reference
Glioblastoma stem cells (GSCs)	↑	—	[101]
Lung cancer (NSCLC)	↑	—	[92, 94]
Ovarian carcinoma	↑	—	[85]

Bladder cancer	—	↑	[121]
Chronic myeloid leukemia	—	↑	[139]
Colon adenocarcinoma	—	↑	[135]
Colorectal cancer	—	↑	[118]
Fibrosarcoma	—	↑	[131]
Gastric cancer	—	↑	[138]
Hepatocellular carcinoma (HCC)	—	↑	[127]
Kaposi sarcoma	—	↑	[128]
Lung cancer (NSCLC)	—	↑	[120, 122, 134, 137]
Melanoma	—	↑	[123, 131]
Oral squamous cell carcinoma	—	↑	[125]
Pancreatic cancer	—	↑	[119, 124, 126, 133]

Breast cancer	↑	↑	[86]
Cervical cancer	↑	↑	[152]
Chronic myeloid leukemia	↑	↑	[136]
Esophageal squamous carcinoma	↑	↑	[82]
Gallbladder cancer	↑	↑	[79]
Glioblastoma	↑	↑	[100, 155]
Glioblastoma stem cells (GSCs)	↑	↑	[100]
Hepatoma	↑	↑	[152]
Lung cancer (NSCLC)	↑	↑	[87, 89, 152]
Malignant B lymphocytes	↑	↑	[154]
Mammosphere stem cells (MSC)	↑	↑	[102]
Multiple myeloma	↑	↑	[153]
Neuroblastoma	↑	↑	[90, 91]
Ovarian carcinoma cells	↑	↑	[156]
Prostate cancer	↑	↑	[88]
Renal cancer	↑	↑	[117]
